# Framing visual roll-motion affects postural sway and the subjective visual vertical

**DOI:** 10.3758/s13414-016-1150-3

**Published:** 2016-06-30

**Authors:** Astrid J. A. Lubeck, Jelte E. Bos, John F. Stins

**Affiliations:** 1 Research Institute MOVE, Faculty of Behavioural and Movement Sciences, VU University Amsterdam, De Boelelaan 1091, 1081 HV Amsterdam, Netherlands; 2 TNO Perceptual and Cognitive Systems, TNO Soesterberg, Kampweg 5, 3769DE Soesterberg, The Netherlands

**Keywords:** Postural control, Subjective visual vertical, Visually induced motion sickness, Perception of verticality, Optic flow, Roll-motion

## Abstract

Effects of visual roll-motion on postural sway and the subjective visual vertical (SVV) often is studied using mechanical devices, whereas electronic displays offer cheaper and more flexible alternatives. These devices typically emit and reflect light scattered by the edges of the screen, providing Earth-fixed cues of verticality. These cues may decrease the effects of rotating stimuli, a possibility that has not been studied explicitly before in one experimental design. We exposed 16 participants to a visual dot pattern, either stationary, or rotating in roll, that was or was not surrounded by a visible Earth-fixed reference frame. To eliminate unintended visual cues, the experiment was performed in complete darkness and participants wore neutral density goggles passing only 1% of light. Postural sway was measured using a force platform. SVV measurements were obtained from a visible rod. To monitor the participants, motion sickness severity was obtained with an 11-point rating scale. Results showed that the presence of an Earth-fixed frame significantly decreased the effect of the rotating pattern on postural sway and SVV deviations. Therefore, when studying subjective verticality related effects of visual stimuli, it is imperative that all visual Earth-fixed cues are not just minimized but completely eliminated. The observation that an Earth-fixed frame significantly decreased the effect of the rotating pattern on both postural sway and the SVV points towards a common neural origin, possibly involving a neural representation of verticality. Finally, we showed that an electronic screen can yield similar effect sizes as those taken from the literature using mechanical devices.

**Electronic supplementary material:**

The online version of this article (doi:10.3758/s13414-016-1150-3) contains supplementary material, which is available to authorized users.

## Introduction

In order to identify correctly “up” and “down” with respect to the Earth gravitational field, it has been suggested that our central nervous system uses a neural representation of the Earth-vertical (Mach, 1875; Bourdon, 1906; Barra et al., 2010). Such a neural representation can be described as a vector with a magnitude and orientation. In this experiment, we focus on the orientation of this vector, further referred to as the neural representation of verticality.

Previous research has shown that this neural representation is essential to orient behaviour with respect to the Earth vertical (Angelaki, Shaikh, Green, & Dickman, 2004; Barra et al., 2010; Pérennou et al., 2008). For the most veridical neural representation of verticality, it is continuously updated with integrated cues from the visual, vestibular, and somatosensory systems (Bos, Bles, & Groen, 2008; Merfeld, Zupan, & Peterka, 1999; Zupan & Merfeld, 2003). Visual Earth-fixed cues are thought to aid a veridical representation, whereas visual tilt or motion, without accompanying physical motion, is thought to cause a deviation thereof (Bos et al., 2008; Mittelstaedt, 1983; Zupan & Merfeld, 2003). By measuring behaviours that arguably use the neural representation of verticality, effects of such visual stimuli can be quantified.

Studies using static tilted stimuli, such as a tilted furnished room or just a tilted luminous square, have shown that postural control, i.e. “the act of maintaining, achieving or restoring a state of balance during any posture or activity” (Pollock, Durward, Rowe, & Paul, 2000), is affected whilst viewing a tilted frame (Guerraz et al., 2001; Isableu, Ohlmann, Cremieux, & Amblard, 1997; Pavlou et al., 2011). Besides postural control, subjective visual vertical (SVV) estimates of a straight line are “attracted” by the tilted frame, a phenomenon known as the Rod-and-Frame effect (Dichgans, Brandt, & AP, 1978; Guerraz et al., 2001; Guerraz, Poquin, & Ohlmann, 1998; Isableu, Gueguen, Fourré, Giraudet, & Amorim, 2008; Pavlou, Davies, & Bronstein, 2006; Witkin & Asch, 1948a, b).

Similar postural excursions and SVV deviations can be induced by viewing a dynamic visual stimulus, e.g., a pattern rotating in roll as used in the Rod-and-Disc test (Agarwal et al., 2012; Guerraz et al., 2001; Pavlou et al., 2011). Viewing a stimulus in roll is thought to cause a tilt of the neural representation of verticality, in this case in the direction of the stimulus rotation (Bles, Bos, de Graaf, Groen, & Wertheim, 1998; Bos et al., 2008; Bos & Bles, 2002; Mittelstaedt, 1983; Zupan & Merfeld, 2003). This can be inferred from both postural sway and the SVV, typically both deviating in the direction of stimulus rotation (Dichgans, Held, Young, & Brandt, 1972; Guerraz et al., 2001; Pavlou et al., 2011; Tanahashi, Ujike, Kozawa, & Ukai, 2007).

Besides serving a scientific interest, rotating visual stimuli have been proposed to be beneficial in the clinical context of vestibular rehabilitation. The idea behind this approach is that repeated exposure to visual motion can reduce the severity of disturbances caused by visual motion in certain vestibular patients (Guerraz et al., 2001; Pavlou, Bronstein, & Davies, 2013; Pavlou, Lingeswaran, Davies, Gresty, & Bronstein, 2004; Pavlou, 2010; Riecke & Schulte-pelkum, 2013). This kind of therapeutic intervention can be facilitated by using electronic screens instead of previously used mechanical devices (Pavlou, 2010; Pavlou et al., 2012, 2013; Roberts, Bronstein, & Seemungal, 2013; Vitte, Sémont, & Berthoz, 1994).

A potential confounding factor when using such techniques is that most displays provide unintended visible Earth-fixed cues of verticality. Light emitted and reflected by objects in the “completely darkened” experimentation room can result in unintended visual cues, which may affect the representation of verticality. A particular source of reflection concerns the edges of the display itself. As shown by the Rod-and-Frame effect, these Earth-fixed cues may influence our neural representation of verticality, thus possibly affecting the variables of interest. If, for example, a rotating stimulus failed to elicit an effect, this might have been due to the (unintended) presence of visible Earth-fixed cues instead of another assumed reason. Despite this issue having been recognized and efforts having been described to reduce or eliminate these Earth-fixed cues (Dyde, Jenkin, & Harris, 2006; Guerraz & Bronstein, 2008; Isableu et al., 2008), the question remains how influential these Earth-fixed cues actually are.

We therefore performed an experiment with the main objective to scrutinize the effect of a visible Earth-fixed cue—in this case a rectangular visual reference frame—on two variables that are arguably controlled or at least affected by the neural representation of verticality during exposure to visual roll-motion. The two variables are postural sway and the SVV. In particular, we assume that the influence of visual roll-motion on these variables is smaller when the rotating stimulus is presented together with a static, visible, rectangular frame.

## Method

### Participants

Sixteen healthy young adults participated voluntarily after signing an informed consent form. Due to technical difficulties during the experiment, data of two participants were discarded, leaving 14 participants for further analyses. Participants were students at the Faculty of Human Movement Sciences of the VU University, 8 males and 6 females, with a mean age of 20.6 years (standard deviation [*SD*] = 1.4 years). Ethical approval was provided by the Ethics Committee of the same faculty in accordance with the Declaration of Helsinki.

### Apparatus and stimulus

Participants, in a completely darkened room, were exposed to stimuli shown on a 40-inch TV-screen (Samsung LE40B620; 88 × 50 cm; width × height) in high-definition resolution (1920 × 1080 pixels) with a monitor refresh rate of 60 Hz. To block the edges of the screen and eliminate other unwanted visual Earth-fixed cues, two precautions were taken. First, the edges of the screen were covered using a removable low reflective cardboard cover, leaving a circular viewing area with a radius of 25 cm. Second, participants wore neutral-density (ND) glasses that passed only 1% of light. As a result, all Earth-fixed cues made visible by scattered light were eliminated, while maintaining visibility of the actual stimulus. Participants stood at a distance of 70 cm from the screen, yielding a Field of View (FoV) of 40 × 40°.

**Fig. 1:**
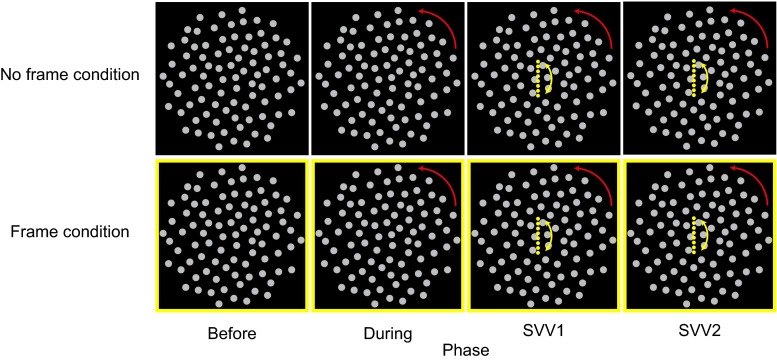
Overview of the procedure. The rows represent the two conditions: the No Frame (NF) and Frame (F) condition, respectively. The columns represent the phases during each trial: before, during, SVV1, and SVV2 phase, respectively. Each condition consisted of three stimulus types. In one stimulus type, the dot pattern remained stationary. In the other two stimulus types, the dot pattern rotated clockwise and counter clockwise from the during phase until the end of the stimulus type. Examples of the counter-clockwise stimulus type are depicted. During the SVV1 and SSV2 phases, participants were asked to adjust a rod to match their subjective visual vertical, which appeared once rotated leftward of the Earth-vertical and once rightward of the Earth-vertical in each trial. For the postural sway, only data from the before and during phases were included

The stimulus consisted of two components: a computer-generated dot pattern and a computer-generated frame. The dot pattern, as shown in Fig. 1, consisted of 88 nonoverlapping dots, each dot subtending a FoV of approximately 1.5°. In case the pattern was rotating (see procedures, below), the pattern rotated with an angular velocity of 30°/s (one revolution per 12 s). The pattern enclosed the complete circular viewing area (area left uncovered by the cardboard). In a subset of trials, the dot pattern was surrounded by a 50-cm yellow square frame with an edge width of 0.24 cm. When the frame was present, the cover with the circular viewing area was removed. The TV screen was carefully positioned Earth-horizontal to ensure unbiased SVV measurements (see below).

### Variables

During exposure, participants were standing on a custom-made, 1- × 1-m strain gauge force plate, recording the Centre of Pressure (CoP) at 100 Hz. Participants stood with their arms hanging comfortably by their sides and their feet positioned with the heels together with a 30° angle between the feet as marked on the force plate.

SVV measurements involved aligning a computer generated rod to the perceived vertical. The rod consisted of eight small dots, subtending a visual angle of approximately 5°, which were placed on an imaginary line to minimise quantization visibility (Fig. 1). The orientation of the rod was adjusted by using a handheld keypad with a roughened structure on two buttons, representing clockwise (CW) and counter clockwise (CCW) rotation. The rod was presented with an initial angular offset between 30° and 70° leftward or rightward of the Earth-vertical, and rotated with a speed of 10°/s with incremental steps of 0.17° when a button was pressed. Participants were allowed to correct the rod to align it with their perceived vertical as long as the rod was visible. Participants held the keypad in both hands when the rod appeared on the screen for SVV measurements and kept it in their right hand in-between SVV measurements.

Finally, it has been suggested that rotating visual stimuli can be provocative with respect to visually induced symptoms, such as disorientation (Bos et al., 2008; De Graaf, Bles, & Bos, 1998). Therefore, for observational purposes we included VIMS as a third variable in this experiment. To monitor the occurrence of visually induced symptoms, participants were asked to rate their subjective symptom severity using the misery scale (Bos, MacKinnon, & Patterson, 2005), which is an 11-point scale ranging from 0 to 10. This scale exploits the knowledge that symptoms of nausea are generally preceded by symptoms from the oculomotor and disorientation subscale (Bos et al., 2005). Absence of symptoms is represented by 0; any symptom except nausea is scored between 1 and 5; a score of 6 or higher is given whenever feeling nauseated; 10 represents vomiting.

### Procedure

Before the start of the experiment, participants were informed about the procedure, were familiarized with the MISC, and signed an informed consent form. Participants were asked to put on the ND glasses and to stand on the force plate as depicted. During exposure, participants were instructed to stand as quietly as possible, to look continuously at the centre of the monitor, and to only take a step to prevent falling (which never happened). The experiment consisted of two conditions with three trials each, and each trial was divided into four phases as explained in the following.

The conditions differed as regards the presence of the frame. In the Frame condition (F), the frame was displayed surrounding the dot pattern, whereas in the No Frame condition (NF) the frame was absent (Fig. 1). In the three trials, the dot pattern did not rotate (still), rotated clockwise (CW), or rotated counter clockwise (CCW). Each trial lasted 108 seconds and was divided into the following four phases. 1) In the “before” phase, the dot pattern was shown motionless for 12 s. 2) In the “during” phase, which lasted 48 s, the pattern remained either stationary or rotated in CW or CCW direction. 3) In the “SVV1” phase, lasting 24 s, a rod appeared with a leftward or rightward angular offset from the Earth-vertical that participants aligned to their perceived vertical. Pattern rotation and presence or absence of the frame remained the same as the during phase. After this phase, the adjusted rod disappeared from the screen. 4) The “SVV2” phase was identical to the previous SVV1 phase, except that a new rod appeared with a random angular offset with an orientation that was to the opposite side of the Earth-vertical. After each trial, participants were asked to rate their misery using the MISC. Between conditions, participants were asked to sit down for a 2-minute rest to avoid fatigue.

Participants were exposed to the two conditions (F and NF) in a random, counterbalanced order. The initial rotation direction (CW or CCW) was randomly chosen at the start of the experiment and then alternated to avoid possible accumulation effects. Each condition always started with the baseline trial (still stimulus type) followed by two trials with rotation (CW and CCW; rotation stimulus type).

### Data analysis

For assessment of postural sway, CoP time series collected in the “before” and “during” phase were included. CoP time series were first filtered using a second order low-pass Butterworth filter with a cutoff frequency of 5 Hz. Due to a small delay between the start of the CoP data collection and start of exposure, the last second of each CoP time series was discarded, leaving a before phase of 11 s and a during phase of 47 s. Because the pattern rotated in the frontal plane, it elicited the largest CoP displacements in the mediolateral (ML) direction. We therefore limited the analyses of the CoP time series to this direction. Anticipating the results, no significant differences were found between CW and CCW trials. For this reason, CoP data for the CCW direction time series were mirrored along the ML-axis, and an average CoP-ML time series was calculated. We thus calculated postural sway parameters for the two stimulus types (still and rotation) with averaged (over CW and CCW rotation trials) values for the rotation stimulus type.

Two parameters were calculated from these CoP time series for each phase: the mean moving window standard deviation (MWSD), and the lean. The MWSD was calculated by taking SDs for 1-s, nonoverlapping time windows of 100 data points each, which were then averaged, providing a MWSD for each phase (Riley, Stoffregen, Grocki, & Turvey, 1999). The MWSD was favoured over the standard deviation taken over the entire phase because of substantial CoP shifts in ML direction—anticipated and observed—to which the MWSD is insensitive. The lean was defined as the average positional shift of the CoP in ML direction in the during phase relative to the mean CoP position in the before phase. Positive values denote a postural deviation into the direction of rotation and negative values denote a deviation in the opposite direction.

The SVV deviation was defined as the angular difference between the orientation of the adjusted rod and the Earth-vertical in degrees. Anticipating the results, no differences were found between initial rod offset orientation angles with respect to the Earth-vertical (CW and CCW) and the CW and CCW rotation trials. Therefore, first an average was calculated over the SVV deviations collected in the SVV1 and SVV2 phases for each trial. Second, the SVV deviations for CCW rotation trials were mirrored along the vertical axis, and an average over the CW and CCW trials was calculated. Positive values denote a deviation into the direction of rotation and negative values denote the opposite.

### Statistical analysis

IBM SPSS Statistics 21 was used for statistical analysis. The assumption of normality for postural sway parameters and SVV deviations was checked with Shapiro-Wilk tests and inspection of q-q plots. Effects of phase (before, during), stimulus type (still, rotation), and frame (F, NF) on the MWSD were examined with a 2 × 2 × 2 repeated measures (RM) ANOVA. To determine whether the lean in the during phase was significantly different from the before phase, for all combinations of stimulus type and frame, four one-sample *t* tests with the test value set at 0 (representing the mean position in the before phase) were performed. Second, a 2 × 2 RM ANOVA was conducted on the lean values to study effects of frame and stimulus type.

The statistical analysis of the SVV employed the same factors as the analysis of the lean. First the SVV deviations were compared to the Earth vertical (0°) using four one-sample *t* tests. Second, to analyze effects of frame and stimulus type, a 2 × 2 RM ANOVA was used. Significant main effects were followed up with pairwise comparisons. Significant interaction effects were followed up using planned contrasts and inspection of interaction plots. To determine effect size partial eta-squared (*η* ^*2*^) was calculated. The significance level was set at 0.05.

Anticipating the results, no differences were found between CW and CCW trials and an average over these trials was computed for the MISC rates. Effects of stimulus type (still, rotation) and frame (F, NF) on MISC rates were studied using four nonparametric Wilcoxon signed-ranks tests with a Bonferroni correction (*p* = 0.0125*)*.

## Results

### Postural sway

**Fig. 2:**
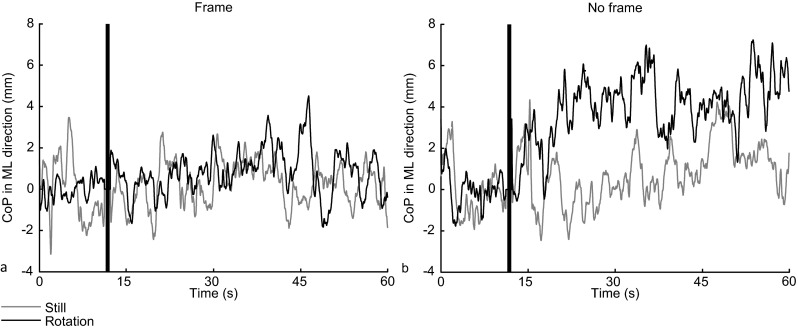
The grand averaged (GA) CoP traces in ML direction for the still stimulus type and rotation stimulus type (averaged over CW and CCW after mirroring of CCW data). (**a**) GA CoP in the Frame condition. (**b**) GA CoP in the No-Frame condition. The black vertical bar represents the transition from the before phase to the during phase

Figure 2 shows the grand averaged CoP for the F and NF condition. This figure reveals that the CoP clearly deviated into the rotation direction in the NF condition whilst rotation and not in the F condition. In other words, when exposed to CW and CCW roll-motion in the absence of a frame, participants gradually shifted their CoP to the right and left, respectively.

**Fig. 3:**
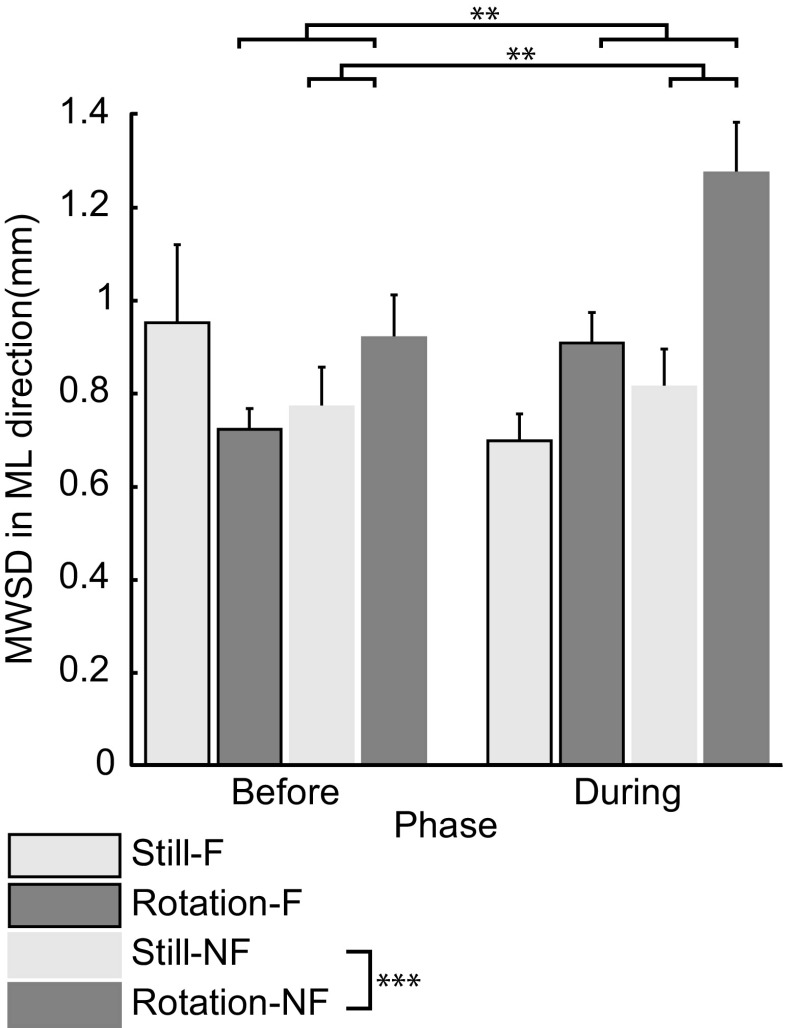
Mean MWSD (in mm, ±SE) for the frame and no frame conditions. “Before” indicates the phase before rotation. “During” indicates the phase with rotation but only for the rotation stimulus types. Significant differences at *p* < 0.01 and *p* < 0.0001 are indicated with ** and *** respectively

Effects of the frame and stimulus type were statistically present in our two CoP parameters. Figure 3 shows the mean MWSDs before and during rotation, separately for the still and rotation stimulus types, and for the F and NF conditions. Statistical analysis revealed three significant two-way interaction effects for the MWSD (all statistics are reported in Table 1). As expected, the interaction between phase and stimulus type showed that only in case of rotation (rotation stimulus type) the MWSD increased significantly in the during phase (*Mean* = 1.095 mm, *SE* = 0.072 mm) compared with before (*Mean* = 0.825 mm, *SE* = 0.056 mm), *p* = 0.008. More importantly, the significant interaction effect between phase and frame revealed that during rotation, the MWSD was significantly increased compared with the before phase in the NF condition, whereas no such increase was present in the F condition, *p* = 0.002. A significant influence of the frame also was revealed by the significant interaction between frame and stimulus type. The MWSD was significantly elevated (*Mean* = 1.101 mm, *SE* = 0.084 mm) in case of rotation compared with a stationary pattern for the NF condition (*Mean* = 0.796 mm, *SE* = 0.074 mm) but not in the F condition, *p* < 0.0001.

**Table 1:** Results RM ANOVA’s for the MWSD, lean and SVV

Source	*Df*	*F*	*η*_*p*_^*2*^	*p*
MWSD				
Frame	1	2.66	0.17	0.127
Phase	1	3.35	0.21	0.09
Stimulus type	1	4.37	0.057	0.25
Phase × Frame**	1	9.64	0.43	0.008
Frame × Stimulus type **	1	15.87	0.55	0.002
Phase × Stimulus type ***	1	31.20	0.71	<0.0001
Phase × Frame × Stimulus type	1	.264	0.02	0.616
Lean				
Frame*	1	5.65	0.303	0.034
Stimulus type	1	3.94	0.23	0.07
Frame × Stimulus type	1	3.57	0.215	0.08
SVV				
Frame***	1	24.65	0.66	<.0001
Stimulus type **	1	17.39	0.57	.001
Frame × Stimulus type **	1	18.56	0.59	.001
Error	13			

*p < 0.05; **p < 0.01; ***p < 0.0001.

**Fig. 4:**
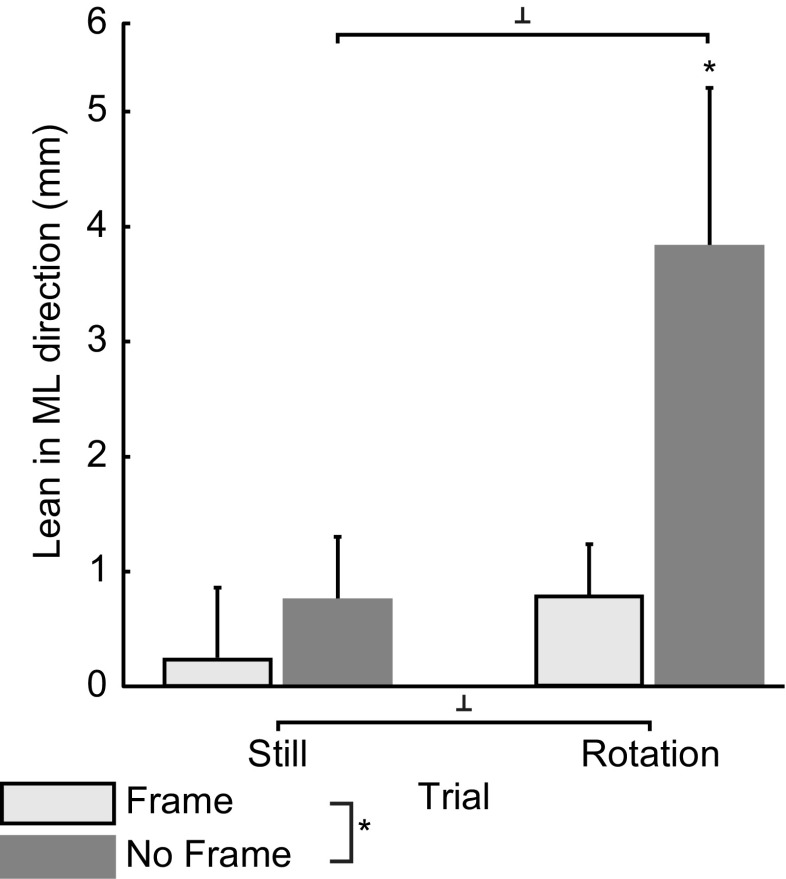
Mean lean in ML direction (in mm, ±SE) for the frame and no frame conditions. Positive values indicate a lean in the direction of rotation. Negative values indicate a lean in the direction opposite to the rotation direction (if there was rotation). Significant differences at *p* < 0.05 are indicated with *. Trends at *p* < 0.08 are indicated with ^⊥^

Figure 4 shows the average lean for both stimulus types and conditions. One-sample *t* tests revealed that the lean was only significantly different from 0 (i.e., the before phase) during rotation in the NF condition, implying that the CoP shifted into the rotation direction compared to before rotation, *t*(13) = 2.82, *p* = 0.014, *r* = 0.61. The RM ANOVA revealed that rotation and the interaction between rotation and frame affected the lean in a similar way as the MWSD but failed to reach significance (Table 1). Only the frame significantly suppressed the lean (Table 1).

### Subjective visual vertical

**Fig. 5:**
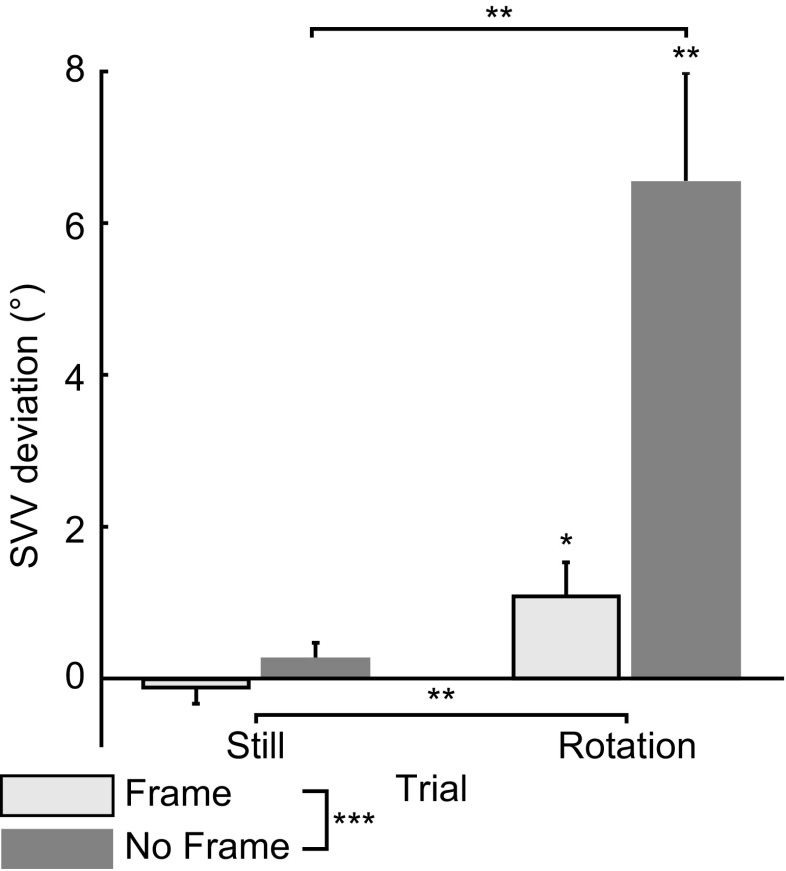
Mean SVV deviation (in degrees ± SE) for both conditions. Positive values indicate a SVV deviation in the direction of rotation. Negative values indicate a SVV deviation in the direction opposite to the rotation direction (if there was rotation). Significant differences at *p* < 0.05, *p* < 0.01, and *p* < 0.0001 are indicated with *, **, and ***, respectively

Figure 5 shows mean SVV deviations in degrees (^o^) for all stimulus types and conditions. First, one-sample *t* tests showed that only in case of rotation the SVV deviated significantly from 0 in the direction of rotation, *t*(13) = 3.16, *p* = 0.02, *r* = 0.66 and *t*(13) = 4.64, *p* = 0.0005, *r* = 0.62 for the F and NF conditions respectively. Second, the RM ANOVA revealed that (1) presence of a frame significantly reduced the SVV deviation; (2) rotation of the dot pattern significantly increased SVV deviation; and (3) rotation had a significantly larger effect on SVV deviations in the NF condition compared with the F condition (Table 1). In other words, the SVV deviated significantly more from the Earth-vertical when the pattern rotated without a frame (*Mean* = 6.57°, S*E* = 1.42°) than during rotation with a frame (*Mean* = 1.13°, *SE* = 0.425°), *p* = 0.001.

### Visually induced symptoms

**Fig. 6:**
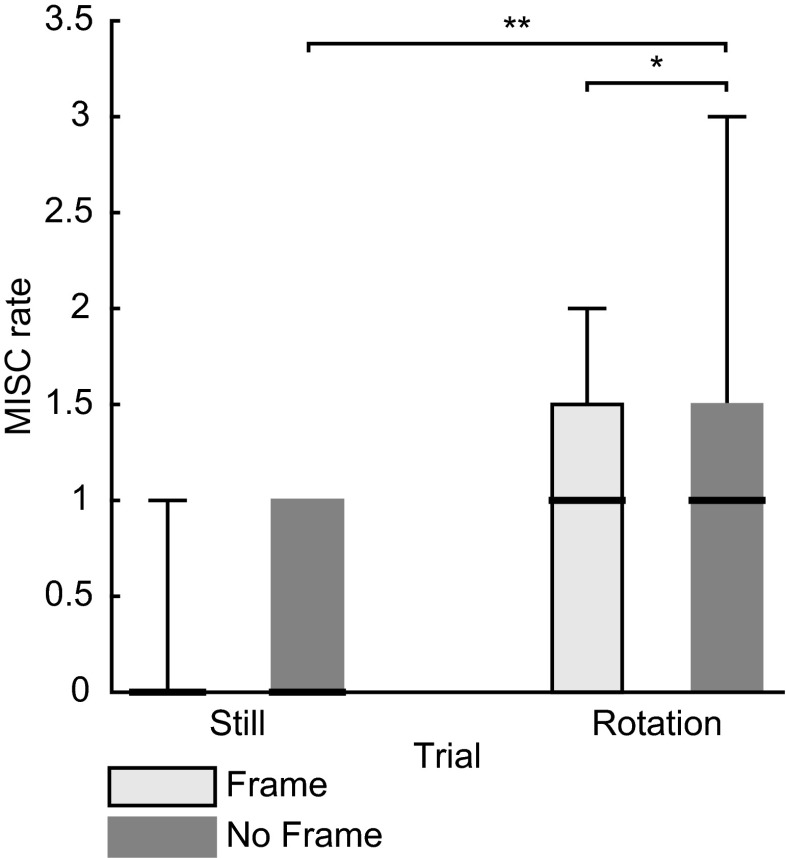
Boxplots of the MISC rates for both conditions and stimulus types. Solid black lines indicate the median MISC rate. Significant differences at *p* < 0.05 and *p* < 0.01 are indicated with * and ** respectively

The maximum reported MISC rate was 3 and was obtained in the rotation trials in the NF condition. This implies that the stimuli did cause some symptoms, such as disorientation, but no nausea. In the NF condition, participants reported higher MISC rates after the rotation trials (*Mdn* = 1) compared with the still trial (*Mdn* = 0), *Z* = 2.82, *p* = 0.005, *r* = 0.75. In the F condition, MISC rates after the rotation trials (*Mdn* = 1) were higher than after the still trial (*Mdn* = 0) but did not reach significance, *Z* = 2.40, *p* = 0.016 (note the Bonferroni corrected significance level of 0.0125; Fig. 6). Moreover, the MISC rates reported after the rotation trial in the NF condition were significantly higher compared with the MISC rates reported after the rotation trial in the F condition, *Z* = 2.52, *p* = 0.012, *r* = 0.67.

## Discussion

We investigated the combined effect of an Earth-fixed visual reference frame and visual roll-motion on postural sway and the SVV. As hypothesized, both variables revealed that the presence of an Earth-fixed reference frame surrounding the rotating pattern facilitated upright standing (less variability and less lean) and made SVV estimates more accurate (thus, in line with the Earth-vertical).

The finding that both variables were affected by roll-motion and the reference frame suggests that these variables are mediated by one common mechanism. Because of the Earth-fixed orientation of the frame, it could involve a neural representation of verticality. First, we observed that postural sway was less variable (MWSD) and showed smaller excursions (lean) in presence of a visible Earth-fixed reference frame whilst viewing visual roll-motion. Evidence that humans use a neural representation of verticality for postural control has been provided by research in stroke patients with an active lateral tilt of the body (Pérennou et al., 2008). When interpreting the current postural results as a reflection of the neural representation of verticality, they indicate that the neural representation of verticality deviated less when an Earth-fixed cue of verticality was visible.

Second, in line with earlier studies (Dichgans et al., 1972; Guerraz et al., 2001; Pavlou et al., 2011; Tanahashi et al., 2007), it was observed that visual roll-motion caused a significant deviation of the SVV away from the Earth-vertical. Such a deviation is proposed to be possible only if humans possess a neural representation of what is vertical (Bos & Bles, 2002; Mittelstaedt, 1983). The observation that the Earth-fixed reference frame attracted the SVV, comparable to the Rod-and-Frame effect (Dichgans et al., 1978; Guerraz et al., 2001, 1998; Isableu et al., 2008; Pavlou et al., 2006; Witkin & Asch, 1948a, b), also shows that veridical Earth-fixed cues are used to visually estimate what is vertical. Therefore, these findings also support the idea that the SVV is influenced by a reference frame with an Earth-fixed orientation. However, to what extent the Earth-vertical orientation of the reference frame plays a role should be addressed in future research that takes into account multiple orientations. Concluding, these results point towards the involvement of a common neural mechanism that uses sensory information provided by the reference frame, which could include the Earth-vertical orientation.

Interestingly, also the visually induced symptoms were lower when the rotating pattern was surrounded by the Earth-fixed reference frame. Although the observed ratings were low, the MISC rates were increased after exposure to pattern rotation without an Earth-fixed reference frame, whereas MISC rates were not as much increased in the condition with an Earth-fixed reference frame. Visually induced symptoms, including motion sickness, also have been proposed to be driven by an internal model of verticality (Bles et al., 1998; Bos et al., 2008) and may driven together with postural sway and the SVV by a common neural mechanism. Whether all measures are driven by a common neural mechanism or whether visually induced symptoms naturally follow SVV deviations and postural changes without sharing a neural mechanism should be addressed in future research.

In addition, the question how the orientation of the visual reference frame interacts with the Earth gravitational reference frame in the construction of a perception of verticality during exposure to visual roll-motion remains unknown. Based on earlier research using the rod-and-frame test, one could hypothesize that an Earth-fixed orientation would significantly influence the measures of interest more than a fixed reference frame with a random orientation. A further study in which the visual reference frame is dissociated from the Earth gravitational reference frame therefore is suggested.

Compared with two earlier studies that used a Rod-and-Disc apparatus (Guerraz et al., 2001; Pavlou et al., 2011), the use of a 40-inch display yielded similar effects of the rotating pattern on postural sway parameters and SVV deviations. Guerraz et al. (2001) and Pavlou et al. (2011) reported during pattern rotation a mean lean of 5.3 mm and 3.9 mm respectively. In this study, we found a comparable lean of 4.01 mm. Also the mean SVV deviation found in this study (6.57°) is in agreement with the SVV deviations found in earlier studies: 9.78° (Guerraz et al., 2001) and approximately 6.5° (Pavlou et al., 2011). Although the postural excursions and SVV deviations were comparable and the angular speed was identical (30°/s), the Rod-and-Disc apparatus subtended a larger FoV of 60° (Guerraz et al., 2001; Pavlou et al., 2011) than the FoV of 40° subtended by the stimulus in this study. This comparison of results provides support for the claim that screens can be used to induce visual disturbances comparable to the Rod-and-Disc apparatus when certain conditions are met.

However, one should take the FoV into account when comparing results. A rotating stimulus that subtended a FoV of 130°, as used by Dichgans et al. (1972), did cause a significantly larger mean SVV deviation of 15°. Moreover, research on vection strength and duration has shown that they increase with an increasing FoV (Allison, Howard, & Zacher, 1999). Further research should test whether the FoV has a similar effect on postural sway and SVV deviations. Although in this study a clear visible Earth-fixed frame was presented, pilot trials in our lab showed that even a minimal visible frame, i.e., only visible after several minutes of dark adaptation, already showed the limiting effects discussed. Possibly, even a barely visible Earth-fixed cue, e.g., seeing a part of one’s own body through a crack in the goggles or a suboptimally fitting mask for the screen, may already significantly reduce the effect of the rotating visual stimulus. As a consequence, it is of great importance when using light-emitting stimuli with the purpose of inducing visual disturbances that no other Earth-fixed cues are visible. In an experimentation room, this often can only be realized by completely darkening the room in combination with the use of ND-filters and screen covers to minimize the amount of scattered light perceived by the participant to a subthreshold level, as shown in the present study. Outside the experimentation room head-mounted displays, such as the low-cost Oculus Rift (Riecke & Jordan, 2015), may be a good alternative. The Oculus Rift subtends a large FoV of approximately 100° and its foam barriers eliminate all visual Earth-fixed cues (Riecke & Jordan, 2015).

In summary, we observed that postural sway and SVV estimates were influenced by the presence of an Earth-fixed cue surrounding visual roll-motion, pointing towards a common neural origin. From a practical point of view, we provided evidence that a commonly available screen is able to induce significant visual disturbances comparable to mechanical devices, when cues of verticality are properly eliminated. Finally, with this study we showed that when studying subjective verticality related effects of visual stimuli it is imperative that all visual Earth-fixed cues are not just minimized but completely eliminated.

## Electronic supplementary material

Below is the link to the electronic supplementary material.

Supplementary Figure 1 Boxplots of the MISC rates separated for participants starting with the NF condition (left) and with the F condition (right). Solid black lines indicate the median MISC rate. (DOCX 38 kb)
