# Supplementary material for: Framing visual roll-motion affects postural sway and the subjective visual vertical
Source: Atten Percept Psychophys. 2016 Jun 30;78(8):2612–20. doi: 10.3758/s13414-016-1150-3 (PMC5110582; doi:10.3758/s13414-016-1150-3)
Supplement: Supplementary file 1 — Boxplots of the MISC rates separated for participants starting with the NF condition (left) and with the F condition (right). Solid black lines indicate the median MISC rate. (DOCX 38 kb) [file 13414_2016_1150_MOESM1_ESM.docx]

**Supplementary material**


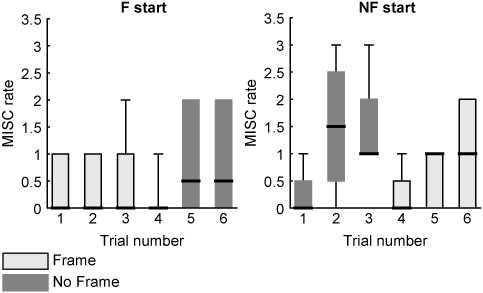


Supplementary figure 1. Boxplots of the MISC rates separated for participants starting with the NF condition (left) and with the F condition (right). The solid black lines indicate the median MISC rate.
